# Prurigo actínico en un centro dermatológico de referencia en Colombia: 108 casos

**DOI:** 10.7705/biomedica.5139

**Published:** 2020-06-30

**Authors:** Andrea Carolina Pardo-Zamudio, Martha Cecilia Valbuena, Héctor David Jiménez-Torres, Claudia Carolina Colmenares-Mejía

**Affiliations:** 1 Facultad de Dermatología, Fundación Universitaria Sanitas, Bogotá, D.C., Colombia Facultad de Dermatología Fundación Universitaria Sanitas BogotáD.C Colombia; 2 Servicio de Fotodermatología, Hospital Universitario Centro Dermatológico Federico Lleras Acosta, Bogotá, D.C., Colombia Hospital Universitario Centro Dermatológico Federico Lleras Acosta BogotáD.C Colombia; 3 Unidad de Investigaciones, Fundación Universitaria Sanitas, Bogotá, D.C., Colombia Fundación Universitaria Sanitas BogotáD.C Colombia

**Keywords:** prurigo, trastornos por fotosensibilidad, fotobiología, rayos ultravioleta, talidomida, Prurigo, photosensitivity disorders, photobiology, ultraviolet rays, thalidomide

## Abstract

**Introducción.:**

El prurigo actínico es una fotodermatosis crónica. Afecta con mayor frecuencia a la población latinoamericana, predomina en mujeres y compromete la piel expuesta al sol, las conjuntivas y los labios.

**Objetivo.:**

Actualizar la información sobre las características clínico-epidemiológicas y el tratamiento de pacientes con prurigo actínico en Colombia.

**Materiales y métodos.:**

Se hizo un estudio de corte transversal que incluyó los registros clínicos de pacientes con prurigo actínico atendidos en el Servicio de Fotodermatología del Hospital Universitario Centro Dermatológico Federico Lleras Acosta entre el 2011 y el 2016, y se describieron sus características demográficas, clínicas e histopatológicas, así como su tratamiento.

**Resultados.:**

Se incluyeron 108 pacientes, el 71,3 % de ellos mujeres y el 28,7% hombres, con predominio de los fototipos III-IV (70 %). La enfermedad se había iniciado durante la primera década de vida en el 66,4% de los casos y el 25 % de los pacientes tenía antecedentes familiares de la enfermedad. Las lesiones predominaban en el rostro (93,5 %), los antebrazos (79,6 %) y el dorso de las manos (70,4 %). También, se documentó compromiso ocular (87,9 %) y de los labios (88,8 %). Se hizo la prueba de fotoprovocación con radiación ultravioleta A en el 25 % de los casos y biopsia cutánea en el 19,4 %. Todos los pacientes se trataron con protección solar química y física. En los casos leves a moderados, se formularon corticoides tópicos (91,7 %) e inhibidores de la calcineurina (65,7 %), y en los graves, talidomida (33,3 %) y pentoxifilina (14,8 %).

**Conclusión.:**

Las características de los pacientes colombianos con prurigo actínico son similares a las reportadas en otros países latinoamericanos: inicio temprano de la enfermedad, predominio en mujeres, compromiso frecuente de conjuntivas y labios, y adecuada respuesta al tratamiento tópico y sistémico.

## Abstract (en)

**Introduction:**

Actinic prurigo is a chronic photodermatosis. It affects the Latin American population more frequently, predominantly women, and involves the sun-exposed areas of the skin, conjunctiva, and lips.

**Objective:**

To update the information on the clinical-epidemiological characteristics and treatment of patients with actinic prurigo in Colombia.

**Materials and methods:**

We conducted a cross-sectional study including the medical records of patients with actinic prurigo treated in the Photodermatology Service of *Hospital Universitario Centro Dermatol*ó*gico Federico Lleras Acosta* between 2011 and 2016. We described the demographic, clinical, histopathological, and treatment characteristics of the patients.

**Results:**

We included 108 patients, 77 (71.3%) were women and 31 (28.7%) men, mainly with phototypes III-IV (70%). The disease had begun during the first decade of life in 66.4% of the cases and 25% of the patients had a family history with the condition. The lesions predominated on the face (93.5%), forearms (79.6%), and back of the hands (70.4%). Ocular (87.9%) and lip (88.8%) involvement was also documented. A photo-provocation test with UVA was performed in 25% of the cases and skin biopsies in 19.4%. Physical and chemical photoprotection was indicated in all patients. Mild to moderate cases were treated with topical corticosteroids (91.7%) and calcineurin inhibitors (65.7%) while severe cases received thalidomide (33.3%) and pentoxifylline (14.8%).

**Conclusion:**

The characteristics of actinic prurigo patients in Colombia are similar to those reported in other Latin American countries: early onset of the disease, predominance in women, frequent involvement of conjunctiva and lips, and adequate response to topical and systemic treatment.

El prurigo actínico, denominado así por Londoño en 1961 ^1^, es una fotodermatosis idiopática crónica que ocurre principalmente en población amerindia y en mestizos (mezcla de amerindios y europeos) ^2^^,^^3^. La patogenia podría ser secundaria a una reacción de hipersensibilidad retardada (T CD4+) a autoantígenos inducidos por la radiación ultravioleta en individuos genéticamente predispuestos ^2^^,^^4^^,^^5^. Dicha reacción involucra la participación de la reacción inmunitaria Th-1 (*T helper*) que ocurre porque la radiación ultravioleta estimula los queratinocitos suprabasales para la producción y liberación del factor de necrosis tumoral alfa (TNF-α) y el interferón gama (IFN-γ), provocando así la apoptosis celular ^4^^,^^6^ y la reacción Th-2 dada por la producción de interleucinas de tipo 4, 5 y 13 por parte de los linfocitos T activados, los mastocitos y los eosinófilos, así como de IgE por parte de los linfocitos B ^4^^,^^6^.

La predisposición genética se asocia con la presencia de diversos alelos HLA de clase I, como el Cw4 y Cw3 en Colombia ^7^, A24 y Cw4 en Canadá ^8^ y A28, B16 y B39 en México ^9^ y, especialmente, con alelos de la clase II como el HLA DR4, subtipo DRB1*0407 en la población de Francia ^10^, México ^11^ y Colombia ^12^.

Esta enfermedad aparece generalmente en la primera década de la vida ^2^^,^^13^^,^^14^, aunque también puede aparecer después de los 20 años ^2^. Afecta la piel expuesta a la radiación ultravioleta, así como los labios y las conjuntivas ^2^^,^^3^^,^^15^. Las lesiones cutáneas son polimorfas, muy pruriginosas, localizadas predominantemente en las áreas expuestas a la luz solar, aunque pueden extenderse a las zonas cubiertas ^2^^,^^3^^,^^16^^-^^18^. El diagnóstico es eminentemente clínico, sin embargo, un método útil para respaldarlo es la prueba de fotoprovocación con radiación ultravioleta A y B, ya que permite la reproducción del 75 al 100 % de las lesiones ^2^^,^^3^^,^^19^.

En cuanto al tratamiento, la primera medida es evitar la exposición solar usando protección física y protectores solares de amplio espectro ^2^^,^^15^^,^^16^. Cuando la enfermedad es leve, se utilizan corticosteroides tópicos, inhibidores de la calcineurina, antihistamínicos orales y emolientes ^2^^,^^20^. En caso de exacerbaciones, los pulsos cortos de corticosteroides orales han demostrado ser eficaces ^3^^,^^19^. Londoño demostró la utilidad de la talidomida en el tratamiento del prurigo actínico ^21^ y, desde entonces, se usa en los casos graves de esta fotodermatosis. El efecto secundario más grave es la teratogenicidad ^22^^,^^23^, motivo por el cual las mujeres deben evitar el embarazo durante el tratamiento. En un estudio no controlado, se logró mejoría al mes de tratamiento con pentoxifilina ^24^.

En los estudios en algunos países de Latinoamérica, como Perú y México, así como en Francia, Taiwán, Tailandia, Singapur, Australia, Canadá y el Reino Unido, se han descrito las particularidades sociodemográficas y los hallazgos histopatológicos de los pacientes con prurigo actínico y, dado que no hay estudios recientes sobre su comportamiento en Colombia, se propuso el presente estudio para establecer dichas características, las manifestaciones clínicas y el tratamiento usado contra la enfermedad en la población de estudio.

## Materiales y métodos

Se hizo un estudio observacional descriptivo de corte transversal en el Servicio de Fotodermatología del Hospital Universitario Centro Dermatológico Federico Lleras Acosta en Bogotá (Colombia), entre enero de 2011 y diciembre de 2016.

Se incluyeron los pacientes con diagnóstico clínico de prurigo actínico atendidos en la institución mediante un muestreo no probabilístico, consecutivo y a conveniencia a partir del registro de las historias clínicas. Se excluyeron aquellos registros clínicos con información incompleta en las variables de interés, así como los que no estaban digitalizados en el sistema electrónico Dinámica™. En todos los casos, la recolección de la información sobre las variables se extrajo de los registros electrónicos de las historias clínicas.

Las variables de estudio incluyeron la información sociodemográfica (edad, sexo, zona de residencia, ocupación y antecedentes familiares), las características clínicas (comportamiento de la enfermedad con la exposición solar y el calor, fototipo, evolución, edad de inicio de la enfermedad, localización anatómica de las lesiones, sintomatología, localización y tipo de lesiones), los resultados de la prueba de fotoprovocación y la biopsia cutánea o de labios, y el tratamiento (medidas de protección, corticoide tópico y sistémico, inhibidores de la calcineurina, talidomida, antihistamínicos o pentoxifilina). 

### Análisis estadístico

Se utilizó el paquete estadístico Stata 13.0™. Las variables cualitativas se reportaron como frecuencias absolutas y relativas, y las cuantitativas, como mediana y rangos intercuartílicos (RIC). Se reportaron, asimismo, los datos perdidos de cada variable de interés.

### Consideraciones éticas

El estudio fue aprobado por el Comité Institucional de Ética en Investigación y se ajustó a las pautas del Consejo de Organizaciones Internacionales de las Ciencias Médicas (CIOMS) y los criterios de Ezekiel Emmanuel. Según la normatividad nacional e internacional, esta fue una investigación sin riesgo, no hubo intervenciones y no se violó la privacidad de los pacientes.

## Resultados

**Figura 1:**
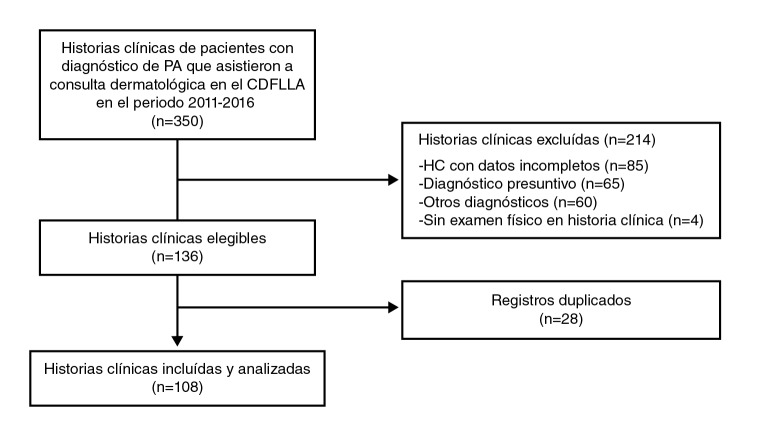
Flujograma de la selección de historias clínicas

El proceso de selección de los registros clínicos se presenta en la figura 1. De los 108 pacientes incluidos en el estudio, la mayoría (71,3 %) fueron mujeres. La mayor parte de los pacientes eran naturales y residentes de regiones urbanas cercanas a Bogotá (2.650 msnm) con altitudes por encima de los 1.000 msnm; en cuanto a la ocupación, la más frecuente fue la de estudiante. Se reportaron antecedentes en familiares de primer grado en el 25 % de los pacientes y solo en dos (1,9 %) de los casos no se contaba con dicha información. El fototipo más común (41,7 %) fue el IV, seguido del III, el II y el V, con 28,7, 14,8 y 1,9 %, respectivamente, en tanto que en el 13 % no se reportaba información. En el cuadro 1, se presentan las características sociodemográficas de la población de estudio.

CDFLLA: Centro Dermatológico Federico Lleras Acosta

**Cuadro 1:** Características sociodemográficas de la población con prurigo actínico (n=108)

Variable	n	(%)
Edad (años)*	33	(19,44)
Sexo		
Femenino	77	(71,3)
Masculino	31	(28,7)
Lugar de residencia		
Cundinamarca	96	(88,9)
Boyacá	10	(9,3)
Tolima	1	(0,9)
Santander	1	(0,9)
Altitud de lugar de residencia (msnm)		
<1.000	1	(0,9)
>1.000	107	(99,1)
Zona de residencia		
Urbana	99	(91,7)
Rural	8	(7,4)
Sin información	1	(0,9)
Ocupación		
Estudiante	25	(23,2)
Otras **	15	(13,9)
Vendedor al aire libre	12	(11,1)
Ama de casa	9	(8,3)
Agricultor	4	(3,7)
Conductor	4	(3,7)
Industria	4	(3,7)
Constructor	3	(2.8)
Comerciante-ventas	2	(1,9)
Sin información	30	(27,8)

^*^ Mediana (RIC)

^**^ Otras: profesor de educación física, jardinero, cocinero, oficina, veterinario, guardería de perros, trabajador en minas

El inicio del prurigo actínico fue temprano en 71 (66,4 %) casos, con una mediana de edad de 8 años (RIC: 7 a 13 años) y, tardío, en 36 (33,6 %) pacientes, con una mediana de edad de 35 años (RIC: 28,5 a 39,5 años).

El tiempo medio de aparición de las lesiones después de la exposición solar fue de 6 horas (rango: 2 a 23 horas), y la mayoría (78,7 %) de los pacientes refirió exacerbación de la enfermedad con la exposición solar. El 50 % de ellos manifestó exacerbación de las lesiones con la radiación ultravioleta A filtrada a través de las ventanas. De los 72 pacientes que en algún momento de la vida visitaron algún lugar de menor altitud, 30 reportaron mejoría, otros 30 empeoraron, en tanto que 12 no notaron cambios en las lesiones cutáneas y en 33 casos dicha información no se registró en la historia clínica.

**Figura 2:**
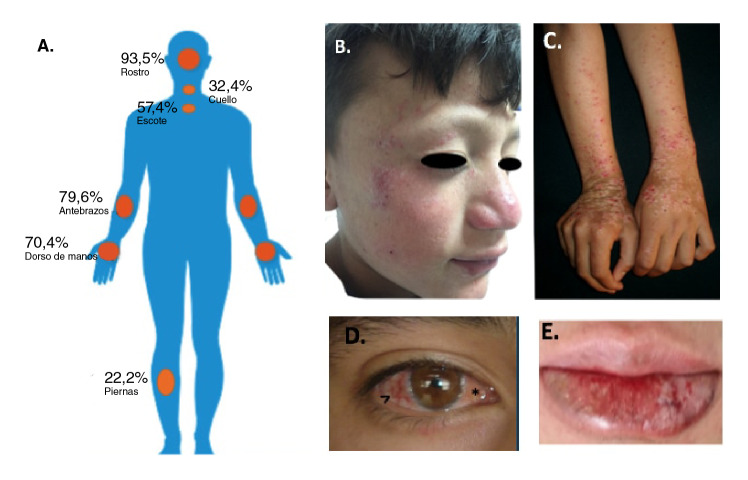
Localización de las lesiones de prurigo actínico en la población de estudio. **A)** Frecuencia de las lesiones por áreas anatómicas. **B)** Detalle de las lesiones en el rostro. **C)** Compromiso del dorso de las manos (pápulas liquenificadas, excoriaciones, costras y cicatrices hipocrómicas). **D)** Lesiones oculares: conjuntivitis en banda (>) y pingüécula (*). **E)** Compromiso de labio inferior; se evidencian placas eritematosas y descamativas y algunas fisuras.

El prurito fue el síntoma principal (96 %) y las lesiones eran permanentes en todos los pacientes; el compromiso anatómico general se describe en la figura 2A. La afectación del rostro se dio en el 93,5 %, siendo las mejillas y el área malar las zonas más afectadas (86,1 %), seguidas del dorso nasal (60,2 %), la frente (51,9 %), las orejas (28,7 %), el mentón (28,7 %), la punta nasal (25 %), y la región supraciliar (19,4 %). Las lesiones más comunes fueron las pápulas (84,3 %), las placas (81,5 %), las excoriaciones (79,6 %) y la liquenificación (53,7 %), seguidas de hiperpigmentación (50 %) e hipopigmentación posteriores a la inflamación (35,2 %) y cicatrices (40,7 %) (figuras 2, B y C).

En cuanto al compromiso ocular, el 87,9 % de los pacientes (figura 2D) lo presentaba, siendo la conjuntivitis en banda (73,2 %) y la pingüécula (31,5 %) las manifestaciones más comunes, seguidas del pterigion (29,6 %) y el pseudopterigion (7,4 %). La afectación de los labios ocurrió en el 88,8 % de los casos y el labio inferior fue el más comprometido, con la presencia de placas eritematosas y descamativas (56 %) como característica semiológica principal (figura 2E). El compromiso de las zonas no expuestas a la luz solar estuvo presente en el 15,7 % de los pacientes.

El diagnóstico de prurigo actínico se basó en una minuciosa anamnesis y en los hallazgos clínicos; solo en 27 (25 %) pacientes se efectuaron pruebas de fotoprovocación con radiación ultravioleta A en dosis promedio de 25 J/cm^2^ (RIC: 25-50), las cuales fueron positivas en 21 (77,7 %) pacientes.

Se tomó biopsia cutánea en 21 casos. Los hallazgos histopatológicos más frecuentes en la epidermis fueron acantosis (76,2 %), paraqueratosis (66,6 %) y espongiosis (55,2 %). En la dermis, se observó un infiltrado celular de tipo linfocitario (85,7 %), superficial (75 %) y perivascular (100 %). También, se detectaron vasos ectásicos (11,1 %), colágeno degenerado (100 %) y melanófagos (35,7 %).

El tratamiento usado en la población de estudio se presenta en el cuadro 2. Si bien la talidomida se prescribió a 36 pacientes, solo 28 tomaron el medicamento; la dosis inicial fue de 100 mg/día (85,7 %) seguida de 50 mg/día (14,2 %), con una mediana de duración del tratamiento de un mes y una mediana de mejoría subjetiva del 80 % (rango: 70 a 90 %). La dosis de mantenimiento fue de 200 mg semanales, con una mediana de duración de 3,5 meses (rango: 2 a 5) y una mediana de mejoría subjetiva del 90 %. Se presentaron efectos secundarios en tres pacientes, dos refirieron parestesias en manos y, el otro, vértigo, por lo cual se les suspendió el medicamento y se les reemplazó por pentoxifilina.

**Cuadro 2:** Tratamiento usado en los pacientes con prurigo actínico en un centro de referencia en Bogotá, Colombia (n=108)

Variable	n	(%)
Medidas de protección	108	(100)
*Polypodiumleucotomos* oral	7	(6,5)
Corticoides tópicos	99	(91,7)
Corticoides tópicos (según potencia)		
De gran potencia	62	(57,4)
Alta	5	(4,6)
Media	16	(14,8)
Baja	16	(14,8)
Otros medicamentos		
Corticoides sistémicos	3	(2,8)
Inhibidores de la calcineurina	71	(65,7)
Talidomida	36	(33,3)
Antihistamínicos	30	(27,8)
Pentoxifilina	16	(14,8)

En los pacientes que recibieron pentoxifilina, la dosis más frecuentemente utilizada en los adultos fue de 400 mg cada 12 horas (50 %), seguida de 1.200 mg/día (31,3 %), en tanto que en los niños fue de 20 mg/kg (12,5 %), con una mediana de duración del tratamiento de tres meses y una mediana de mejoría subjetiva del 70 %. Solo cinco pacientes presentaron exacerbación de la enfermedad mientras estaban tomando pentoxifilina, tres de los cuales recibieron talidomida más corticoide tópico, dos, terapia tópica con corticoides o inhibidores de la calcineurina.

## Discusión

En la población evaluada, predominaron las mujeres y el inicio de la enfermedad ocurrió durante la infancia en la mayoría de los casos. Solo una tercera parte de los pacientes requirió otras ayudas diagnósticas, como la biopsia cutánea o la prueba de fotoprovocación. El tratamiento tópico utilizado con mayor frecuencia fue el corticoide, en tanto que el sistémico fue con talidomida y pentoxifilina.

En cuanto al sexo, los hallazgos fueron similares a lo reportado en otras poblaciones latinoamericanas ^25^^-^^28^ y en aquellas de origen caucásico: Escocia ^29^, Australia ^19^, Canadá ^8^ y Francia ^10^, con porcentajes similares o mayores al encontrado en este estudio y predominio de mujeres. Se ha sugerido que el 17 beta-estradiol tiene un efecto protector contra la inmunosupresión ocasionada por la radiación ultravioleta, por lo que las fotodermatosis como el prurigo actínico y la erupción polimorfa lumínica se presentan con mayor frecuencia en las mujeres ^30^. Por el contrario, en los estudios realizados en población asiática, la enfermedad predomina en los hombres (81,8- 94,7 %) ^31^^-^^33^.

El 99,7 % de los pacientes del estudio residían en zonas que se encuentran a más de 1.000 msnm, similar a lo reportado en México ^24^^,^^34^^,^^35^. Sin embargo, también se ha descrito la aparición de la enfermedad en lugares ubicados a altitudes menores, como Lima ^25^ y la Provincia de Trujillo en Perú ^27^, así como en el pueblo indígena chimila, en la Sierra Nevada de Santa Marta en Colombia ^7^^,^^36^, poblaciones estas que residen a nivel del mar.

Tincopa-Wong, *et al*., describen que la mayoría de los pacientes que residen en lugares ubicados a gran altura reporta mejoría de las lesiones cutáneas cuando se desplaza a zonas de menor altitud, pero en ninguna de las publicaciones revisadas se reporta tal mejoría ^18^. En el presente estudio, el 41,6 % de los pacientes presentó mejoría al viajar a regiones de menor altitud, lo que coincide con lo descrito por el autor peruano ^18^; ello se debe a que, en estos lugares, hay menor intensidad de la radiación ultravioleta porque es dispersada y absorbida por la atmósfera en mayor proporción ^37^, Sin embargo, la misma proporción de pacientes empeoró, hallazgo que podría deberse a que Colombia es un país ubicado en una región tropical y muy expuesto a la radiación ultravioleta sin variación estacional, lo que incidiría en la presencia continua de la enfermedad, a diferencia de los países con estaciones, en donde hay mejoría en el otoño y en el invierno ^2^^,^^13^^,^^18^.

La frecuencia del antecedente en familiares de primer grado fue semejante a la encontrada en la población australiana (28,6 %) ^19^, en tanto que, en otros países latinoamericanos, ha sido inferior, entre 2,85 y 8 % (25,26,35), y en Escocia ^29^ y Canadá ^8^ se reportó en el 46 y el 68 % de los pacientes, respectivamente. Dichos hallazgos pueden coincidir con la asociación entre el prurigo actínico y el HLA de clase II, especialmente el subtipo DRB1*0407, descrita en la población caucásica, la colombiana y la mexicana ^2^^,^^10^^-^^12^^,^^35^.

En la mayoría de los casos la enfermedad se inicia en la niñez, especialmente durante la primera década de la vida ^10^^,^^25^^,^^29^^,^^35^, y hay un segundo pico de inicio en la edad adulta, con una edad media en la población de Australia ^19^, la de Taiwán ^33^ y la de Singapur ^33^ de 25, 41 y 52 años, respectivamente, lo que es semejante a lo encontrado en el presente estudio.

La relación entre la aparición o exacerbación de las lesiones cutáneas con la exposición solar se ha reportado en Escocia en el 75 % de los casos ^29^, y fue similar en el presente estudio, así como en Asia (6,66-42,1 %) ^31^^,^^38^ y Canadá (92 %) ^8^. La mediana del tiempo de aparición de las lesiones después de la exposición solar fue mayor a la reportada en Escocia ^29^ y Canadá ^8^, donde los pacientes presentaban lesiones cutáneas a los 10 minutos o menos, y a los 93 minutos, respectivamente. Dicha diferencia podría asociarse con las variaciones en la expresividad genética de la enfermedad. Asimismo, la aparición o exacerbación de las lesiones con la radiación que atraviesa las ventanas (radiación ultravioleta A) es semejante a la encontrada en la población escocesa ^29^ y en el pueblo inuit en Canadá ^8^.

El fototipo IV fue el más común, equivalente a lo informado en México ^26^, Perú ^27^^,^^28^ y Asia ^31^^,^^33^^,^^38^. El prurito fue el síntoma principal como se ha reportado en otras poblaciones ^8^^,^^10^^,^^26^^,^^27^^,^^29^^,^^31^^,^^39^. Las zonas anatómicas afectadas con mayor frecuencia fueron similares a las reportadas en la población peruana ^28^, la escocesa ^29^ y la asiática ^19^^,^^33^, en tanto que las características semiológicas de las lesiones cutáneas fueron semejantes a lo encontrado en México ^15^^,^^40^, Perú ^27^, Canadá ^8^, Francia ^10^ y Asia ^32^.

Por otra parte, el compromiso de las zonas cubiertas, como el dorso, el abdomen, los glúteos y los muslos se presentó con una frecuencia similar a aquella en la población caucásica ^10^. El fenómeno de autosensibilización podría explicar la aparición de lesiones cutáneas en las áreas no expuestas a la luz solar en el prurigo actínico ^41^, ya que la radiación ultravioleta estimula la producción de FNT-a, IFN-y y otras citocinas proinflamatorias en los queratinocitos que ejercen su acción de forma local y a distancia, generando apoptosis celular en el foco agudo y en las áreas cubiertas ^42^.

La afectación ocular se presentó en el 87,9 % de los pacientes. En Francia ^10^, Escocia ^29^ y en la población inuit de Canadá ^8^, se ha reportado este compromiso en el 37, 5, 21 y 62 % de los casos, respectivamente, lo que podría deberse a que la conjuntiva está en contacto directo con el medio ambiente y es más propensa a sufrir daños por la exposición prolongada a la radiación ultravioleta. En México, el 30 % de los casos en una serie de niños presentó fotofobia, pingüécula (unilateral o bilateral) y pterigion, lo que evidencia que el compromiso ocular ocurre desde la infancia y puede persistir durante la adultez ^34^. Otro estudio de 40 pacientes demostró el compromiso ocular en el 45 % de ellos ^43^, a diferencia de las poblaciones en Asia ^31^^,^^33^^,^^38^ y Australia ^19^, en las que no hubo compromiso de la conjuntiva.

Al igual que lo reportado en pacientes de México ^35^^,^^39^^,^^40^, Escocia ^29^ y Australia ^19^, en el presente estudio el labio inferior fue el más comprometido por ser anatómicamente más prominente y, por ello, el que recibe más radiación ultravioleta. La afectación de los labios como única manifestación del prurigo actínico se ha descrito en el 27,6 ^35^ al 56 % de los casos ^39^, por lo que puede confundirse con otro tipo de queilitis y retrasar el diagnóstico. Esto contrasta con los reportes en la población asiática, la cual no presenta queilitis ^31^^,^^33^.

La prueba de fotoprovocación con radiación ultravioleta A logró la inducción de las lesiones a las 24 horas de la irradiación en el 77,7 % de los pacientes en quienes se hizo, con lo que se pudo respaldar el diagnóstico evidenciando la sensibilidad a la radiación ultravioleta A en esta fotodermatosis, como se halló en un grupo de pacientes escoceses cuyas pruebas de fotoprovocación con radiación ultravioleta A fueron positivas en el 62 % de los casos evaluados ^29^. En México, Hojyo, *et al*., reprodujeron las lesiones en el 90 % de los pacientes con la radiación ultravioleta A en dosis de 2,5 J/cm^2^ durante 10 días ^3^. Asimismo, el grupo tailandés ^38^ reportó positividad en las pruebas de fotoprovocación con radiación ultravioleta A en el 37 % de los casos, en 40 % de ellos con la combinación de radiación ultravioleta A y B, y en el 13,3 %, con radiación ultravioleta B.

Aunque los hallazgos histopatológicos cutáneos no son específicos en el prurigo actínico, los resultados obtenidos son parecidos a los de otros estudios ^18^^,^^31^^,^^35^^,^^38^. En la biopsia de labio, se ha descrito degeneración vacuolar, ulceraciones y costras, con infiltrado linfocitico dérmico denso y difuso que forma folículos linfoides bien definidos de tipo CD45 en la periferia y células B en su centro ^35^^,^^43^, y presencia de abundantes eosinófilos y algunos melanófagos que no afectan los anexos ni la dermis profunda ^2^^,^^3^^,^^5^^,^^34^^,^^44^. Estos hallazgos, conocidos como queilitis folicular, exhiben una sensibilidad del 74,3 % y una especificidad del 36,4 como factores predictores del prurigo actínico ^34^ y fueron descritos por Vega-Memije, *et al*., en el 63,8 % de los pacientes mexicanos ^35^.

En cuanto al tratamiento, a todos los pacientes se les recomendaron medidas de protección física y uso de protectores solares de amplio espectro con un alto factor de protección solar, así como el uso de corticoides tópicos de gran potencia disminuidos progresivamente, como se ha reportado en otras poblaciones ^8^^,^^10^^,^^19^^,^^29^^,^^31^^,^^35^^,^^38^, con lo cual se obtuvo el adecuado control de la enfermedad hasta en el 83,6 % de los casos ^35^. Para el alivio del prurito, se usó antihistamínico sedante con resultados variables, como ha ocurrido en otros estudios ^8^^,^^10^^,^^29^^,^^31^^,^^38^.

El tratamiento sistémico en la población de estudio incluyó corticoides, talidomida y pentoxifilina. En el caso de las exacerbaciones graves, se prescribieron corticoides orales progresivamente reducidos, como se ha informado en Australia ^19^; en un estudio asiático estos se usaron por vía intramuscular ^31^. Hay reportes del tratamiento con acetónido de triamcinolona intralesional en lesiones recalcitrantes ^32^, especialmente en el dorso de las manos ^38^.

La talidomida se indicó en pacientes con compromiso importante de la enfermedad, pero no todos iniciaron el tratamiento, probablemente debido a que este medicamento no es fácil de conseguir, su costo es alto y, en mujeres y hombres en edad fértil, deben administrarse concomitantemente anticonceptivos dada su teratogenicidad ^22^. Esto, pues se ha demostrado que hacia la cuarta semana de tratamiento las concentraciones de este inmunomodulador son similares en plasma y líquido seminal, por lo cual debe evitarse la concepción y la donación de esperma, y también es obligatorio usar el método de barrera hasta tres meses después de la suspensión del medicamento, tiempo que corresponde a un nuevo ciclo de espermatogénesis ^23^; además, existe la posibilidad de efectos secundarios como la neuropatía. Por lo general, la dosis de inicio es de 100 mg diarios, con mejoría de las lesiones en las primeras cuatro semanas, como se ha reportado en otros estudios ^25^^,^^32^^,^^33^^,^^35^. En Australia, se usó la misma dosis con mejoría, sin embargo, hasta el 50 % de los pacientes presentó urticaria y alteración en la conducción nerviosa, por lo cual se suspendió ^19^.

La pentoxifilina se formuló a los pacientes en que no fue posible emplear la talidomida o en quienes presentaron efectos secundarios a este inmunomodulador; en 16 de estos casos hubo una buena mejoría clínica con dosis menores a las reportadas en un estudio mexicano ^24^, pero el seguimiento fue muy corto.

Entre las limitaciones del estudio cabe mencionar la ausencia de datos sobre algunas variables de interés, pues la recolección de la información se basó en los registros de las historias clínicas y en los reportes de patología. Entre sus fortalezas, no obstante, está la cantidad de pacientes incluidos, así como la información detallada de la anamnesis en cuanto a la reacción a la exposición solar, la presentación clínica y el tratamiento.

Los resultados de este estudio demuestran que el prurigo actínico debe sospecharse en la población colombiana que presente lesiones pruriginosas permanentes en la piel de áreas expuestas a la luz solar, así como compromiso de las conjuntivas y los labios, y se encuentre en la primera década de la vida, aunque sin olvidar la presentación tardía. Debe elaborarse una historia clínica minuciosa, ya que las características clínicas, los antecedentes familiares y el comportamiento de la enfermedad frente a la exposición solar son elementos que ayudan al profesional de la salud a hacer un diagnóstico oportuno. La talidomida sigue siendo el tratamiento de primera línea en los casos graves, a pesar de sus efectos secundarios y teratogénicos en mujeres y hombres en edad fértil.
